# Correspondence

**Published:** 1864-06

**Authors:** 

**Affiliations:** Albany, Linn County, Oregon


### Correspondence.

ALBANY, LINN COUNTY, OREGON,
April 12th, 1864.

J. TAFT:

*Dear Sir*—In making an attempt to comply with your request, by writing a communication for the REGISTER, allow me simply to give you a brief account of the dental profession in the Willamette Valley.

I believe it is now generally acknowledged by all intelligent persons, that dentistry is one of the most important professions, and requires the highest moral and intellectual persons, as well as those most skilled in its manipulations, to succeed well in performing all its different operations in the highest state of perfection to which it has now attained; therefore we ought not to be satisfied with past attainments, or what we now know, but be constantly searching for new truths, and should embrace every opportunity to advance our knowledge. With this view, and believing that the best way to become wise is by associating with those wiser than ourselves, I submit the following:

The Willamette Valley is a large and beautifully situated valley, and is well adapted to agricultural pursuits, stock raising, &c., &c. It is composed of the following named counties, towns, &c. (Census of 1863.)

| Names of Counties. | Population. | Principal Cities and Towns. |
|---|---|---|
| Lane, | 4,780. | Eugene City. |
| Linn, | 6,772. | Albany, Harrisburg, Brownsville, &c. |
| Marion, | 7,088. | Salem, Sublimity and Silverton. |
| Clackamus, | 3,466. | Oregon City. |
| Benton, | 3,074. | Corvallis. |
| Polk, | 3,625. | Dallas and Independence. |
| Yamhill, | 3,245. | Lafayette and McMinnville. |
| Washington, | 2,801. | Hillsborough and Forest Grove. |
| Multnomah, | 4,150. | Portland. |
| Clatsop, | 498. | Astoria. |

Settled through these different counties there are some where in the neighborhood of fifteen dentists and would-be dentists. Of this number we find a part good, honest, upright men, who make excellent citizens, and are willing to do all that lies in their power to advance the interests of their profession, their country and their God. These men are of that number who have entered the profession with a full knowledge of the responsibilities and the practice, toil and perseverance that would be required to perform all the different operations in their highest state of perfection. Would there were many more such men in every profession and calling in life; but there are so few here that we have been unable as yet to form into any kind of an association to look after the interests of the dental profession. We find another class here who have emigrated to this country from different parts of the world; they make great pretences, say they have studied and practiced dentistry in the old country, or some of the eastern cities, or have graduated at the Cincinnati, or some other College of dental surgery, when really, if the truth were revealed, they never saw a dental college, and never perhaps even visited one of these cities in their lives. Such persons may humbug society, support fast horses, dispense with a tolerable good quantity of bad whiskey and lager beer, but care very little for their profession, only so far as it may enable them to shuffle the almighty dollar into their pockets.

There is another class, who (as one told me the other day, when I asked him why he did not get some books, take some of the journals and post himself up), have learned it all (as he said he had), before commencing to practice. Of still another class, I think of nothing that would better illustrate them and their doings than for each one to have a sign with a duck painted on it as emblematic of their good qualities and virtues; for as you are aware, one of these fowls are invariably going quack, quack, quack. What a blessing it would be if this, or any other country could dispose of all such dentists; yet we find there was a Judas of old, and I suppose we can not expect any thing better in the future, than that we will always, to some extent, not only in our own profession alone, but in all professions and callings in life, be troubled with impostors, hypocrites and traitors. Then what remains for us to do is to endeavor to educate the people in such a manner that they will shun these impostors as they would one of those vile, poisonous reptiles called "copperheads."

Yours, &c., A LOVER OF TRUTH.

